# Lonicerin attenuates house dust mite-induced eosinophilic asthma through targeting Src/EGFR signaling

**DOI:** 10.3389/fphar.2022.1051344

**Published:** 2022-12-23

**Authors:** Zhenan Deng, Xuefei Zhang, Junjie Wen, Xiaojing Yang, Lingna Xue, Changxing Ou, Jianjuan Ma, Hongrui Zhan, Xiaomin Cen, Xuliang Cai, Yu Zhang, Riken Chen, Qingling Zhang

**Affiliations:** ^1^ State Key Laboratory of Respiratory Diseases, Department of Pulmonary and Critical Care Medicine, Guangzhou Institute of Respiratory Health, National Clinical Research Center for Respiratory Disease, National Center for Respiratory Medicine, The First Affiliated Hospital of Guangzhou Medical University, Guangzhou, China; ^2^ Department of Rehabilitation Medicine, Zhujiang Hospital, Southern Medical University, Guangzhou, China; ^3^ Department of Pediatric Hematology, Affiliated Hospital of Guizhou Medical University, Guiyang, China; ^4^ Department of Rehabilitation, The Fifth Affiliated Hospital of Sun Yat-sen University, Zhuhai, China; ^5^ Department of Critical Care Medicine, First Affiliated Hospital of Guangzhou Medical University, Guangzhou, China

**Keywords:** asthma, eosinophil, lonicerin, network pharmacology, molecular docking, src, EGFR

## Abstract

Eosinophilic asthma is the predominant phenotype of asthma, and although these patients are sensitive to glucocorticoid therapy, they also experience many side effects. Lonicerin is a kind of bioflavonoid isolated from the Chinese herb *Lonicera japonica* Thunb, which has anti-inflammatory and immunomodulatory effects. The aim of this study was to elucidate the effects of lonicerin on eosinophilic asthma and its potential mechanisms. Here, we established a house dust mite (house dust mite)-induced eosinophilic asthma model in BALB/c mouse, and evaluated the effects of lonicerin on it. Our results showed that lonicerin significantly reduced airway hyperresponsiveness the number of inflammatory cells (especially eosinophils) and the elevation of interleukin (IL)-4, IL-5, IL-13 and eotaxin in bronchoalveolar lavage fluid (BALF) supernatants of mice. Additionally, lonicerin also eminently blunted inflammatory infiltration and mucus secretion, as well as mRNA levels of Mucin 5AC (MUC5AC) in lung tissue. Furthermore, results of network pharmacology and molecular docking revealed that Src kinase and epidermal growth factor receptor may be the potential targets responsible for the effects of lonicerin. Finally, *in vivo* experiments confirmed that lonicerin inhibited activation of the Src/EGFR pathway by decreasing their phosphorylation. Taken together, the present study demonstrated that lonicerin could suppress HDM-induced eosinophilic asthma in mice through inhibiting the activation of Src/EGFR pathway, which also provides a basis for further research as a new potentially therapeutic agent for eosinophilic asthma and its underlying mechanisms in the future.

## 1 Introduction

Asthma is a well-known chronic respiratory disease that affects approximately 1%–18% of the population worldwide (GINA, 2022), and it is driven by airway inflammation, which triggers biological changes such as mucus production, airway wall remodeling and bronchial hyperresponsiveness (Papi et al., 2018). It was gradually discovered that asthma is highly heterogeneous and can be classified into various phenotypes or endotypes, such as type 2-high (mainly eosinophilic asthma) and type 2-low (mainly neutrophilic or paucigranulocytic asthma) (Hammad and Lambrecht, 2021). A recent study showed that the eosinophilic phenotype accounted for up to 76.8% of severe asthma patients in China (Zhang et al., 2022). Currently, Inhaled corticosteroids are widely considered to be the most basic treatment for asthma, effectively controlling symptoms and preventing exacerbations. However, long-term corticosteroid use may have certain side effects on general health (Costello and Cushen, 2020). Therefore, the quest for some safe and effective asthma treatment drugs is always on the way for researchers.

Traditional Chinese Medicine (TCM) has distinctive effects in the treatment of chronic diseases and has been widely recognized as a core component of complementary and alternative medicine (Newman and Cragg, 2020). *Lonicera japonica* Thunb (Called Jinyinhua in Chinese) is a medicine food homology herb with a wide range of pharmacological effects, such as anti-inflammatory, antibacterial, antiviral, antioxidant, etc. (Shang et al., 2011; Ge et al., 2022). Notably, *Lonicera japonica* extract was found to alleviate OVA-induced asthma or allergic rhinitis in mice (Hong et al., 2013; Lin et al., 2019; Li et al., 2021). Lonicerin is a kind of bioflavonoid isolated from the Chinese herb *Lonicera japonica* Thunb and has been shown to possesses anti-inflammatory and immunomodulatory functions in some studies (Lee et al., 1995; Lee and Han, 2011; Lee et al., 2022). However, there are no studies reporting whether lonicerin has a therapeutic effect on asthma.

Network pharmacology is the product of rapid developments in the combination of bioinformatics, systems biology and integrative pharmacology, which can explore the relationships between compound-gene-disease networks and predict the potential mechanism of TCM for the treatment of corresponding diseases (Li and Zhang, 2013; Zhang et al., 2019). In this study, based on the fact that house dust mite (HDM) as a common allergen can more closely mimic clinical asthma (Gorska, 2018), we first investigated the effect of lonicerin on HDM-induced eosinophilic asthmatic mice. Next, network pharmacology was applied to uncover the potential targets of lonicerin for asthma treatment. Finally, molecular docking analysis and experiments were applied to validate the reliability of the key targets.

## 2 Materials and methods

### 2.1 Chemicals and reagents

**FIGURE 1:**
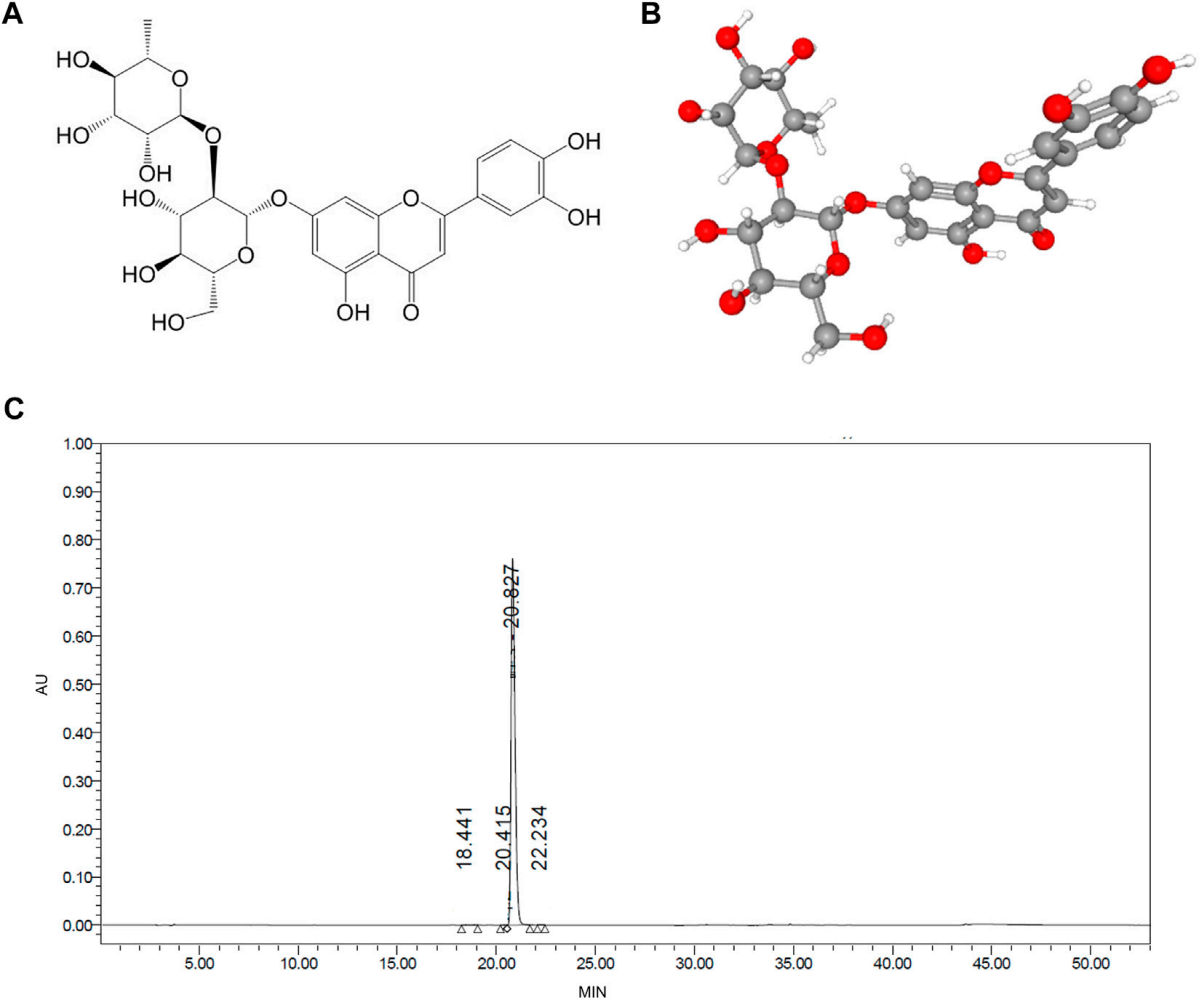
The molecular structure and purify of lonicerin. **(A,B)** 2D and 3D molecular structure were obtained from the PubChem database **(C)** The purify of lonicerin was detected by HPLC.

Lonicerin was purchased from Push Bio-Technology Co., Ltd (Chengdu, China) and the purity (>98%) was confirmed by high-performance liquid chromatography (HPLC) (Figure 1C). HDM was purchased from Greer Laboratories (Lenoir, NC, United States). Methacholine was purchased from Sigma-Aldrich (Sigma-Aldrich China, Shanghai, China). Enzyme‐linked immunosorbent assay (ELISA) kits were purchased from MultiSciences Biotech, Co., LTD. (Hangzhou, China). Hematoxylin-eosin (HE), periodic acid Schiff (PAS) and Congo red staining kits were supplied from Servicebio Technology Co., Ltd. (Wuhan, China). The antibodies including anti-p-Src, anti-p-EGFR, anti-Src, anti-EGFR and anti-β-actin were obtained from Abcam (Cambridge, MA, United States).

### 2.2 Establishment of a mouse model of eosinophilic asthma and drug treatment

**FIGURE 2:**
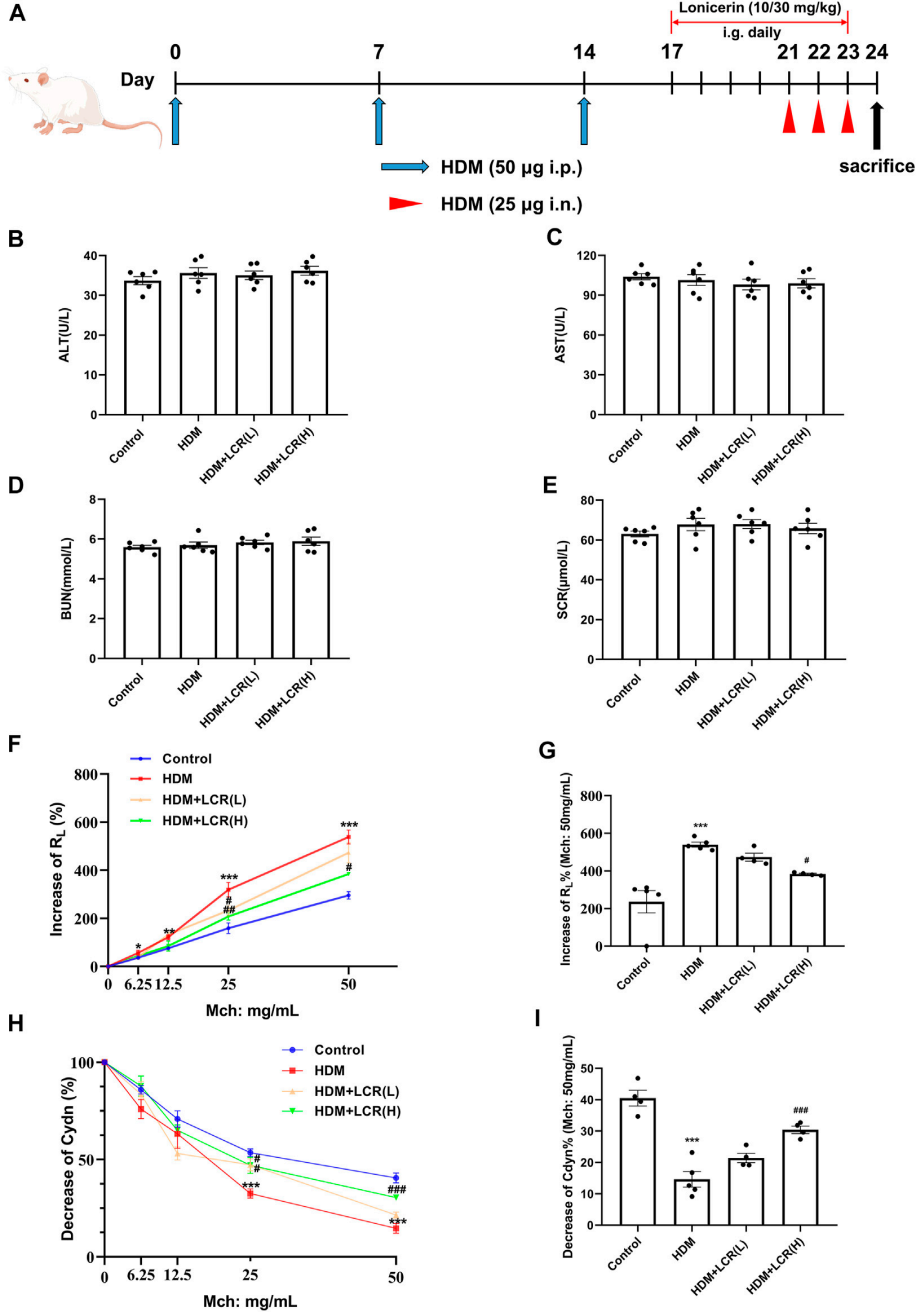
Effects of lonicerin on liver and kidney function and AHR in a mouse model of HDM-induced eosinophilic asthma. **(A)** Asthma model establishment and treatment protocol as described in *Materials and Methods*. **(B–E)** the levels of AST, ALT, BUN and SCR in serum detected by Automatic Biochemistry Analyzer. (*n* = 6). **(F)** Increase of R_L_%. **(G)** Changes in R_L_ to 50 mg/ml of Mch dose. **(H)** Decrease of Cdyn%. **(I)** Changes in Cdyn to 50 mg/ml of Mch dose. (*n* = 4–5). Data are expressed as mean ± SEM. Differences between groups were determined with one‐way ANOVA followed by the Tukey’s *post hoc* test. *p < 0.05, **p < 0.01, ***p < 0.001 vs. Control group; #*p* < 0.05, ##*p* < 0.01, ###*p* < 0.001 vs. HDM group.

Female BALB/c mice aged 6–8 weeks and weighing approximately 20 g were purchased from Youda (Guangzhou) Biotechnology Co. Mice were housed in an SPF facility and treated as described in Figure 2A. Mice were randomly assigned to four groups (*n* = 6 for each group) (GINA, 2022): Normal control group (Papi et al., 2018); HDM-induced eosinophilic asthma model group (Hammad and Lambrecht, 2021); 10 mg/kg low dose lonicerin intervention group: HDM + Lonicerin (L) (Zhang et al., 2022); 30 mg/kg high dose lonicerin intervention group: HDM + Lonicerin (H). The dosage of lonicerin was referred to the previous study (Gu and Sun, 2020; Lv et al., 2021). All of the above groups were modeled by HDM, except for the control group. On days 0, 7, and 14, mice were sensitized with 50 µg HDM by intraperitoneal (i.p) injection and then were challenged intranasally (i.n.) with 25 µg HDM from day 21 to day 23 for three consecutive days. Lonicerin was administered intragastrically (i.g.) from day 17 to day 23. Lonicerin was dissolved with 5% DMSO. During the administration, the control and model groups were given equal amounts of 5% DMSO. Animal experimental procedures were approved by the Animal Care and Use Committee of the First Affiliated Hospital of Guangzhou Medical University.

### 2.3 Measurement of airway hyperresponsiveness

On day 24, Buxco’s modular and invasive system (DSI-Buxco, St. Paul, MN, United States) was used to detect bronchial provocation test in mice, and the changes in airway resistance (R_L_) and lung dynamic compliance (Cdyn) were recorded to characterize airway hyperresponsiveness. Briefly, the anesthetized mice were tracheally intubated and exposed to methacholine (Mch) at sequentially increasing concentrations (0, 6.25, 12.5, 25 and 50 mg/ml) to evaluate lung function.

### 2.4 Serum collection and drug toxicity detection

Serum was extracted from a blood sample by centrifugation at 12,000 rpm for 15 min and stored at −80°C. Four indicators of liver and kidney function in serum: alanine aminotransferase (ALT), aspartate aminotransferase (AST), blood urea nitrogen (BUN) and serum creatinine (SCR), were detected by Chemray-120 Automatic Biochemistry Analyzer (Rayto Life and Analytical Sciences Co., Ltd., China).

### 2.5 Bronchoalveolar lavage fluid (BALF) analysis

After the trachea was dissected, a sterile tube was inserted and lavaged twice with 1 ml sterile phosphate buffered saline (PBS). The collected BALF were centrifuged at 4°C (500 g, 10 min). The total cell count in BALF was calculated and the number of different inflammatory cells was determined by cytospin stained with HE. The levels of IL-4, IL-5, IL-13 and eotaxin in BALF supernatants were determined by ELISA kits according to the manufacturer’s protocol.

### 2.6 Lung histology

The right lung was fixed with 4% paraformaldehyde and embedded in paraffin, and then 4-μm tissue sections were prepared. Referring to previous criteria, HE and PAS staining was used to measure inflammatory infiltration and mucus secretion in the airways, and masson staining was utilized to assess collagen deposition. Since eosinophils are not easily distinguished from other inflammatory cells in mouse lungs, they were detected by Congo red staining. The statistics of these three staining results were as described in previous studies (Huang et al., 2017; Deng et al., 2020; Xue et al., 2021). Briefly, inflammation scores are as follows: 0, no inflammatory cells; 1, occasional inflammatory cells; 2, only one layer of inflammatory cells around most bronchi or blood vessels; 3, 2 layers of inflammatory cells around most bronchi and blood vessels; 4, more than 2 layers of inflammatory cells around most bronchi or blood vessels. In addition, PAS scores are based on the percentage of PAS-positive goblet cells: 0, <5%; 1, 5%–25%; 2, 25%–50%; 3, 50%–75%; 4, >75%.

### 2.7 Immunohistochemistry and immunofluorescence

For immunohistochemistry, the lung sections were blocked with 5% goat serum after antigen retrieval, and then immunostained with corresponding antibody (anti-p-Src or anti-p-EGFR) at 4°C overnight, followed by incubation with HRP-conjugated goat anti-rabbit IgG secondary antibody for 30 min at 37°C. At last, the signals detected using a 3, 3′-diaminobenzidine (DAB) peroxidase substrate kit (Servicebio) and then imaged by an Olympus microscope (Mantra, PerkinElmer). The results were analyzed by Image-Pro Plus software and quantified as average optical density (AOD) in the peribronchial area.

For immunofluorescence, the lung sections were blocked in 5% goat serum for 1 h and incubated overnight at 4°C with corresponding antibody (anti-p-Src or anti-p-EGFR). After washing with PBS, lung sections were incubated with Alexa 488 anti-rabbit secondary antibody or Alexa 594 anti-mouse secondary antibody at for 1 h at room temperature. Nuclei were stained with DAPI (4,6-diamidino-2-phenylindole). Photographs were taken by a fluorescence inverted/laser scanning confocal microscope (Leica Imaging Systems). The results were analyzed by Image-Pro Plus software and quantified as fluorescence intensity.

### 2.8 Quantitative real‐time PCR

Total RNA was extracted from lung tissues using TRIzol reagent (Invitrogen). Next, RNA was reverse transcribed into cDNA by HiScript®II Q RT SuperMix for qPCR (Vazyme Biotechnology Co., LTD., Nanjing, China), and then quantification was performed with ChamQ Universal SYBR qPCR Master Mix (Vazyme) on a LightCycler 480 II system (Roche, Switzerland). All procedures refer to the manufacturer’s manual. The levels of GAPDH mRNA were served as an internal reference. Data are expressed as the fold change over the control group by the 2^-△△Ct^ method. The primers (Sangon Biotech, Shanghai, China) are listed in Table 1.

**TABLE 1:** Primer sequences for genes used for qRT-PCR.

Gene		Sequences (5′–3′)
EGFR	Forward	GCC​ATC​TGG​GCC​AAA​GAT​ACC
	Reverse	GTC​TTC​GCA​TGA​ATA​GGC​CAA​T
MUC5AC	Forward	CAG​GAC​TCT​CTG​AAA​TCG​TAC​CA
	Reverse	GAA​GGC​TCG​TAC​CAC​AGG​G
P53	Forward	CCC​CTG​TCA​TCT​TTT​GTC​CCT
	Reverse	AGC​TGG​CAG​AAT​AGC​TTA​TTG​AG
Src	Forward	GAA​CCC​GAG​AGG​GAC​CTT​C
	Reverse	GAG​GCA​GTA​GGC​ACC​TTT​TGT
TNF-α	Forward	CAG​GCG​GTG​CCT​ATG​TCT​C
	Reverse	CGA​TCA​CCC​CGA​AGT​TCA​GTA​G
KDR	Forward	TTT​GGC​AAA​TAC​AAC​CCT​TCA​GA
	Reverse	GCT​CAG​TAT​CAT​TTC​CAA​CCA
GAPDH	Forward	GGC​AAA​TTC​AAC​GGC​ACA​GTC​AAG
	Reverse	TCG​CTC​CTG​GAA​GAT​GGT​GAT​GG

### 2.9 Western blot

Mouse lung tissue samples were lysed with radioimmunoprecipitation assay (RIPA) buffer containing protease and phosphatase inhibitors. The BCA Protein Assay Kit (Fudebio-tech) was used for protein concentration quantification. Proteins were electrophoresed on 10% or 12% SDS-polyacrylamide gels and transferred to PVDF membranes (EMD Millipore). Membranes were blocked with 5% skim milk at room temperature for 1 h and then incubated overnight at 4°C with specific primary antibodies. The membranes were washed three times with Tris-Buffered Saline Tween-20 (TBST) and then incubated with secondary antibodies conjugated with HRP (Abcam) for 1 h at room temperature. At last, the signal was scanned using the Tanon-5200 infrared scanning system (anon Science & Technology Co., Ltd., Shanghai, China) after three washes again and quantified using ImageJ software.

### 2.10 Network pharmacology analysis

#### 2.10.1 Potential targets prediction

The 2D and 3D structures of lonicerin (Figures 1A, B) were obtained from the PubChem (https://pubchem.ncbi.nlm.nih.gov/) and the targets were collected through the SwissTargetPrediction (http://www.swisstargetprediction.ch/).

#### 2.10.2 Disease-related targets screening

The disease-related genes were searched in the GeneCards (https://www.genecards.org/), OMIM (https://www.omim.org/), PharmGKB (https:// www.pharmgkb.org)/, TTD (http://db.idrblab.net/ttd/) and DrugBank (https://go.drugbank.com/), with “bronchial asthma” as the key word. The disease-related targets were obtained by combining with results from the above 5 databases followed by elimination of redundant genes.

#### 2.10.3 Protein-protein interaction (PPI) network construction

The targets were imported into the STRING database (https://string-db.org/) to obtain the PPI network, and the Cytoscape (V3.9.1) was used for network visualization. Finally, the top 10 core targets were constructed through the CytoHubba plug-in.

#### 2.10.4 Gene ontology (GO) and kyoto encyclopedia genes and genomes (KEGG) enrichment analysis

R language software (v4.1.3) was used to analyze the role of the asthma-related targets of lonicerin in gene function and signaling pathways.

### 2.11 Molecular docking analysis

SWISS-MODEL was used to construct the tertiary structure of the top 5 core targets. The 3D structure and free energy surface were created, and the receptor protein was dehydrated using PyMOL software. Hydrogenation and charge calculations were performed using AutoDock software, and AutoDockVina was used for docking to find the optimal conformation.

### 2.12 Statistical analysis

All values are presented as mean ± SEMM. Differences between groups were determined with one‐way ANOVA followed by the Tukey’s *post hoc* test. Statistical comparisons were performed in Prism 8 software (GraphPad, La Jolla, California, United States). Values of *p* < 0.05 were considered statistically significant.

## 3 Results

### 3.1 Effects of lonicerin on liver and kidney function in mice

Firstly, to test whether two concentrations of lonicerin were toxic to mice, the levels of AST, ALT, BUN and SCR in serum were detected, and the results showed that there was no significant difference between four groups (Figures 2B–E), indicating that lonicerin was almost non-toxic.

### 3.2 Lonicerin alleviated AHR in a mouse model of HDM-induced eosinophilic asthma

Compared with the control group, the HDM group showed increases in R_L_ at four concentrations of Mch provocation, and decreases in Cdyn were observed at Mch = 25 and 50 mg/ml, indicating that we successfully established an asthma model with AHR. In addition, both the HDM + LCR L) group (Mch = 25 mg/ml) and the HDM + LCR H) group (Mch = 25 and 50 mg/ml) showed significant decreases in R_L_ as well as increases in Cdyn compared to the HDM group (Figures 2F–I).

### 3.3 Lonicerin reduced the inflammatory cells and type 2-associated cytokines and chemokines in BALF

**FIGURE 3:**
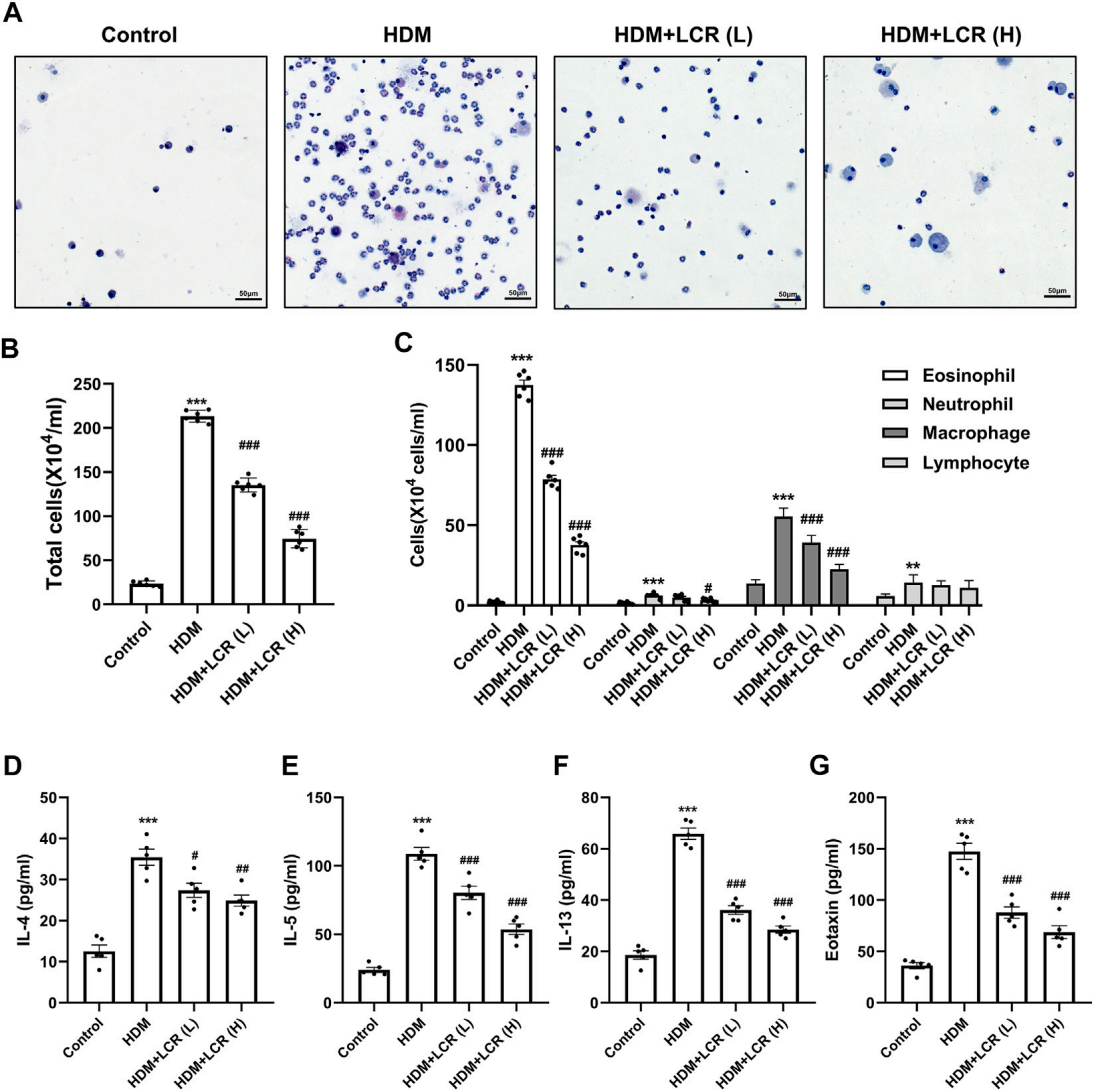
Lonicerin reduced the inflammatory cells and type 2-associated cytokines and chemokines in BALF. **(A)** HE staining was performed to count the cells in the BALF (scale bar, 50 µm). **(B,C)** Total and differentiated inflammatory cells in the BALFs of mice. (*n* = 6). **(D–G)** Levels of IL-4, IL-5, IL-13, and eotaxin in the BALFs supernatant measured by ELISA (*n* = 5). Data are expressed as mean ± SEM. Differences between groups were determined with one‐way ANOVA followed by the Tukey’s *post hoc* test. *p < 0.05, **p < 0.01, ***p < 0.001 vs. Control group; #*p* < 0.05, ##*p* < 0.01, ###*p* < 0.001 vs. HDM group.

As shown in Figures 3A–C, HDM caused an increase in inflammatory cells (mainly eosinophils) in BALF of mice. Compared with the HDM group, the counts of total inflammatory cells, eosinophils, macrophages and neutrophils in the two doses of lonicerin intervention group, especially in the HDM + LCR H) group, were significantly decreased. Next, ELISA was used to detect Type 2-associated cytokines, represented by IL-4, IL-5 and IL-13, and the chemokine eotaxin in the BALF supernatant. The results showed that lonicerin could effectively reduce the elevation of above inflammatory factors induced by HDM (Figures 3D–G).

### 3.4 Lonicerin alleviated inflammatory cell infiltration and mucus hypersecretion in the lungs of asthmatic mice

**FIGURE 4:**
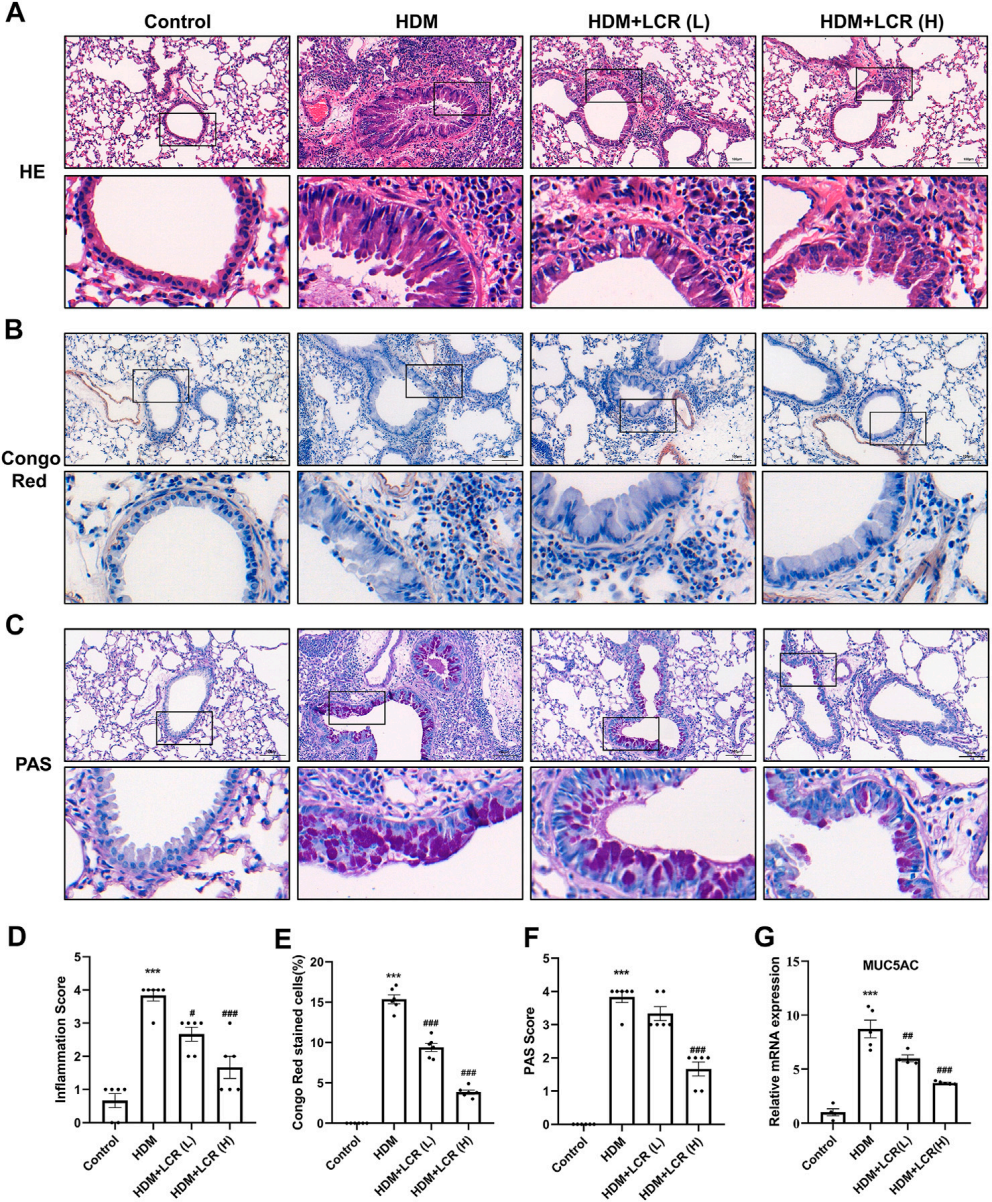
Lonicerin alleviated inflammatory cell infiltration and mucus hypersecretion in the lungs of asthmatic mice. **(A)** HE staining was performed to evaluate airway inflammatory infiltration. **(B)** Lung eosinophils detected by Congo red staining. **(C)** PAS staining was performed to show mucus production (scale bar, 100 µm). **(D–F)** The corresponding quantification histograms of **(A–C)**, respectively. (*n* = 6). **(G)** Detection of MUC5AC mRNA expression in lung tissues of different groups of mice by qPCR. (*n* = 4–5). Data are expressed as mean ± SEM. Differences between groups were determined with one‐way ANOVA followed by the Tukey’s *post hoc* test. *p < 0.05, **p < 0.01, ***p < 0.001 vs. Control group; #*p* < 0.05, ##*p* < 0.01, ###*p* < 0.001 vs. HDM group.

As shown in Figure 4, the results of HE and Congo red staining showed that HDM induced abundant infiltration of peritracheal and perivascular inflammatory cells (especially Congo red-stained eosinophils) and mucosal oedema, which were mitigated by intervention with both concentrations of lonicerin. Similarly, PAS staining score and mRNA expression of MUC5AC, reflecting mucus secretion, were significantly increased in the HDM group, while lonicerin treatment also significantly reduced mucus hypersecretion compared to the HDM group.

In addition, masson staining was used to observe the deposition of collagen fibers in the lungs. Interestingly, our results showed that two concentrations of lonicerin did not significantly reverse HDM-induced collagen fiber production (Supplementary Figure S1).

### 3.5 Identification of the possible targets of lonicerin in the treatment of asthma by network pharmacology and molecular docking analysis

**FIGURE 5:**
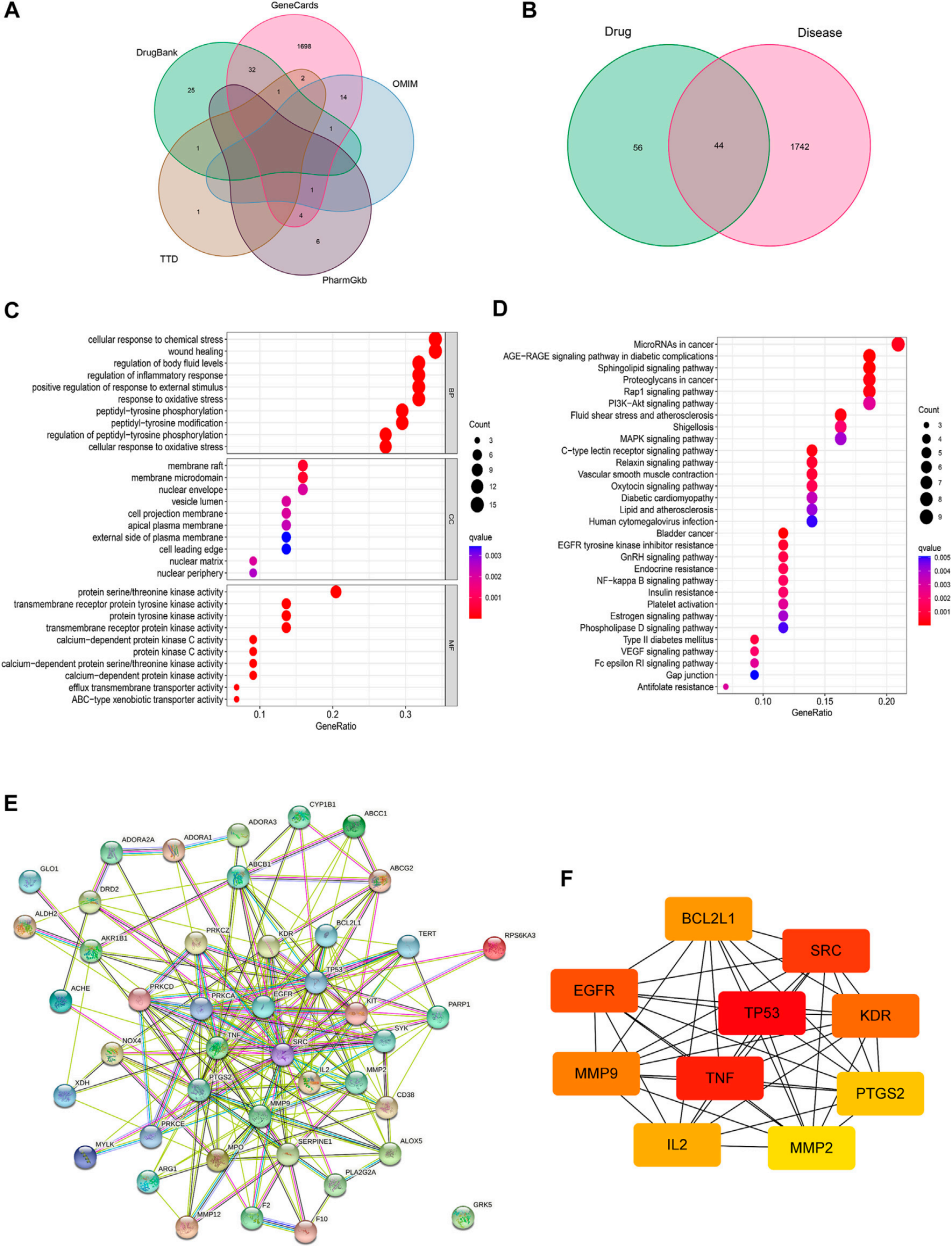
Screening of lonicerin-asthma related target genes. **(A)** Venn diagram of asthma-related genes obtained from various databases. **(B)** Venn diagram of the predicted target genes of lonicerin and asthma-related genes. **(C)** Bubble diagram of GO functional enrichment analysis. **(D)** Bubble diagram of KEGG pathway enrichment analysis. **(E)** PPI network of 44 lonicerin-asthma related target genes. **(F)** Top 10 hub gene analysis.

A total of 1786 asthma-related genes and 100 target genes of lonicerin were obtained after merging and de-duplication by searching in GeneCards, OMIM, PharmGKB, TTD and DrugBank databases (Figure 5A; Supplementary Tables S1, S2). Next, the target genes of lonicerin and asthma-related genes were intersected to obtain a total of 44 lonicerin-asthma related genes (Figure 5B; Supplementary Table S3), which may be potential targets of lonicerin for asthma treatment.

GO enrichment analysis showed that the predicted targets of lonicerin for asthma treatment were involved in biological processes such as cellular responses to chemical stress, wound healing, regulation of body fluid levels, and regulation of inflammatory responses, etc. (Figure 5C). KEGG pathway analysis demonstrated that the predicted targets were enriched in AGE-RAGE pathway, Rap1 pathway, PI3K-Akt pathway, MAPK pathway, EGFR pathway, etc (Figure 5D).

To identify the hub genes and their interactions, we constructed the PPI networks from the 44 targets obtained above in the STRING network platform (Figure 5E). Then, the PPI network was downloaded and imported into Cytoscape software, and the top 10 core targets were sorted and output, including TP53, TNF, SRC, EGFR, KDR, MMP9, BCL2L1, IL2, PTGS2 and MMP2 (Figure 5F).

**FIGURE 6:**
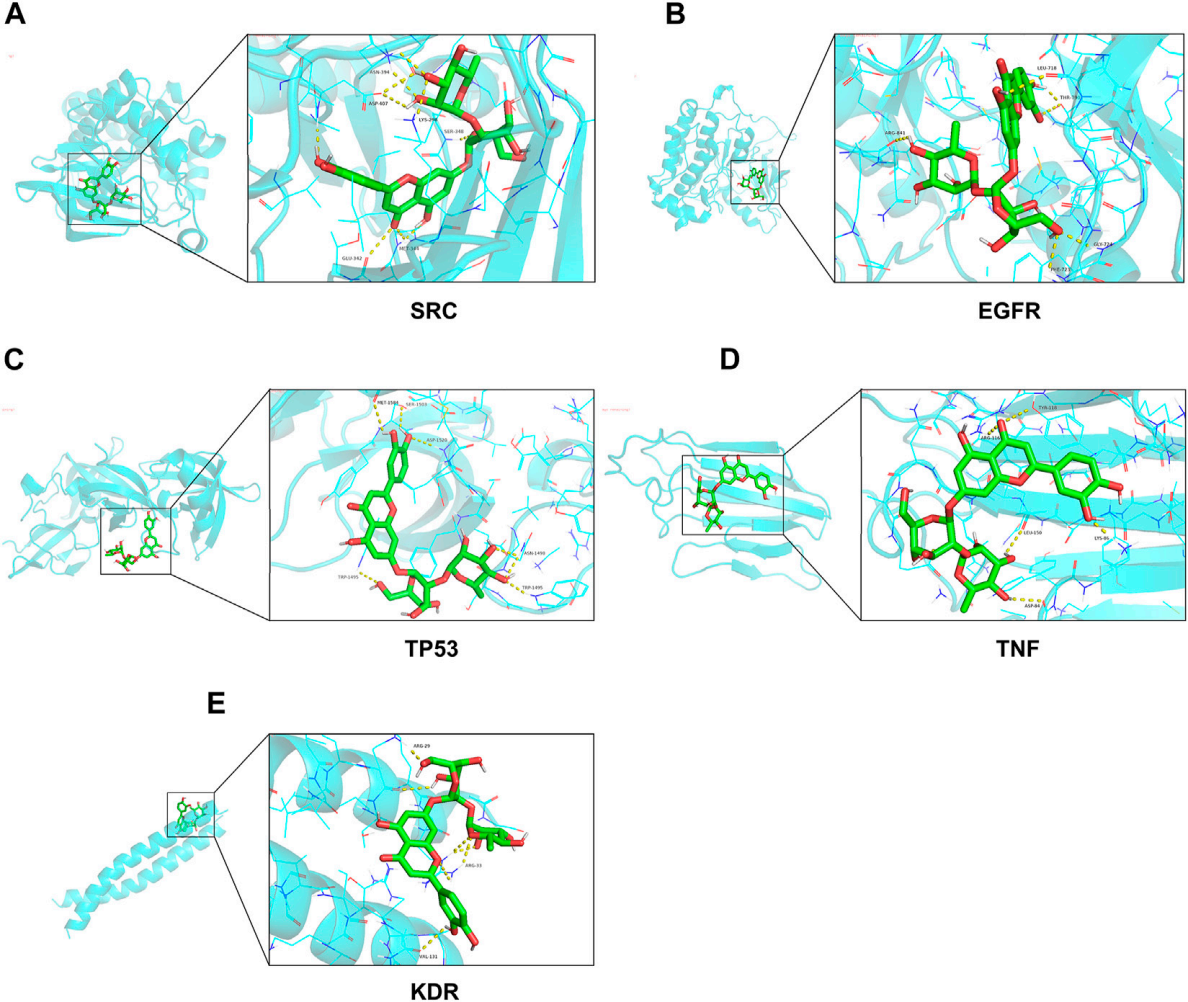
Molecular docking diagrams of lonicerin and top 5 hub genes. **(A–E) (A)** SRC, **(B)** EGFR, **(C)** TP53, **(D)** TNF and **(E)** KDR are shown interacting with a lonicerin molecule.

The results of molecular docking showed that the binding free energy of the top 5 targets obtained above were all less than -5 kcal/mol (Table 2). The visualization of molecular docking was then performed (Figure 6). These results suggested that lonicerin binds well to all of the top 5 targets.

**TABLE 2:** The binding free energy of lonicerin docking with core target molecules.

Gene	Binding free energy (kcal/mol)
SRC	−10.7
EGFR	−8.3
TP53	−7.9
TNF	−7.5
KDR	−7.1

### 3.6 *In vivo* validation of lonicerin for the treatment of asthma by inhibiting the Src/EGFR pathway

**FIGURE 7:**
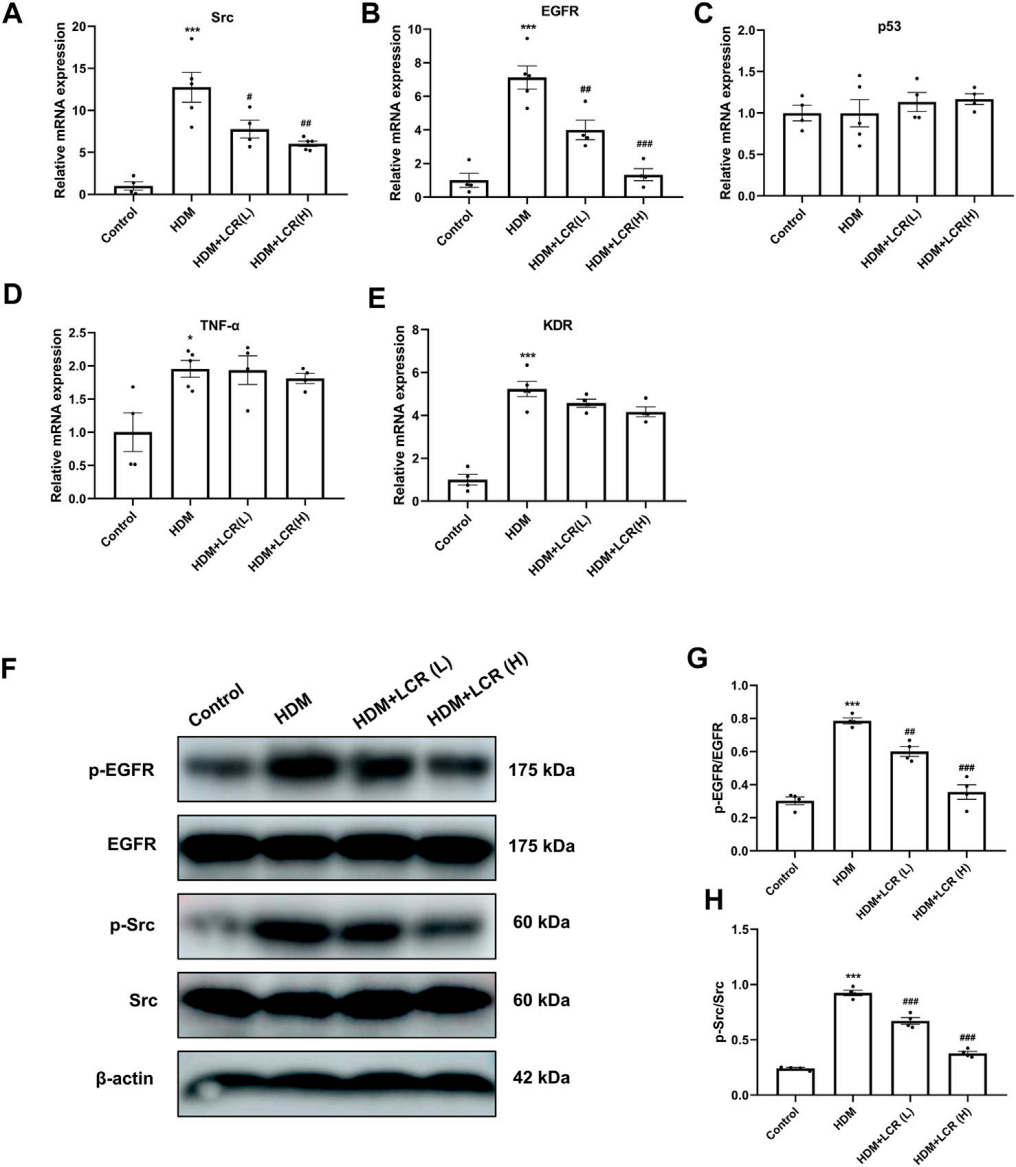
*In vivo* validation of lonicerin for the treatment of asthma by inhibiting the Src/EGFR pathway. **(A–E)** Detection of mRNA expression of Src, EGFR, p53, TNF-α and KDR in lung tissues of different groups of mice by qPCR. (*n* = 4–5). **(F)** Lung tissues from each group were extracted for Western blot to analyse the protein levels of p-EGFR, EGFR, p-Src and Src. **(G,H)** The corresponding quantification histograms of **(F)**. (*n* = 4). Data are expressed as mean ± SEM. Differences between groups were determined with one‐way ANOVA followed by the Tukey’s *post hoc* test. *p < 0.05, **p < 0.01, ***p < 0.001 vs. Control group; #*p* < 0.05, ##*p* < 0.01, ###*p* < 0.001 vs. HDM group.

To verify the reliability of the predicted top 5 target genes, we first measured their mRNA expression by qPCR in different groups of lung tissues, the results showed that the mRNA levels of four genes other than p53 were upregulated in the HDM group compared with the control group. Compared to the HDM group, two concentrations of lonicerin treatment reduced the mRNA levels of Src and EGFR (Figures 7A–E). In addition, Western blot results showed that HDM increased the expression of p-EGFR and p-Src, but had no significant changes in EGFR and Src. The expression of both p-EGFR and p-Src in lonicerin group decreased significantly compared with HDM group (Figures 7F–H).

**FIGURE 8:**
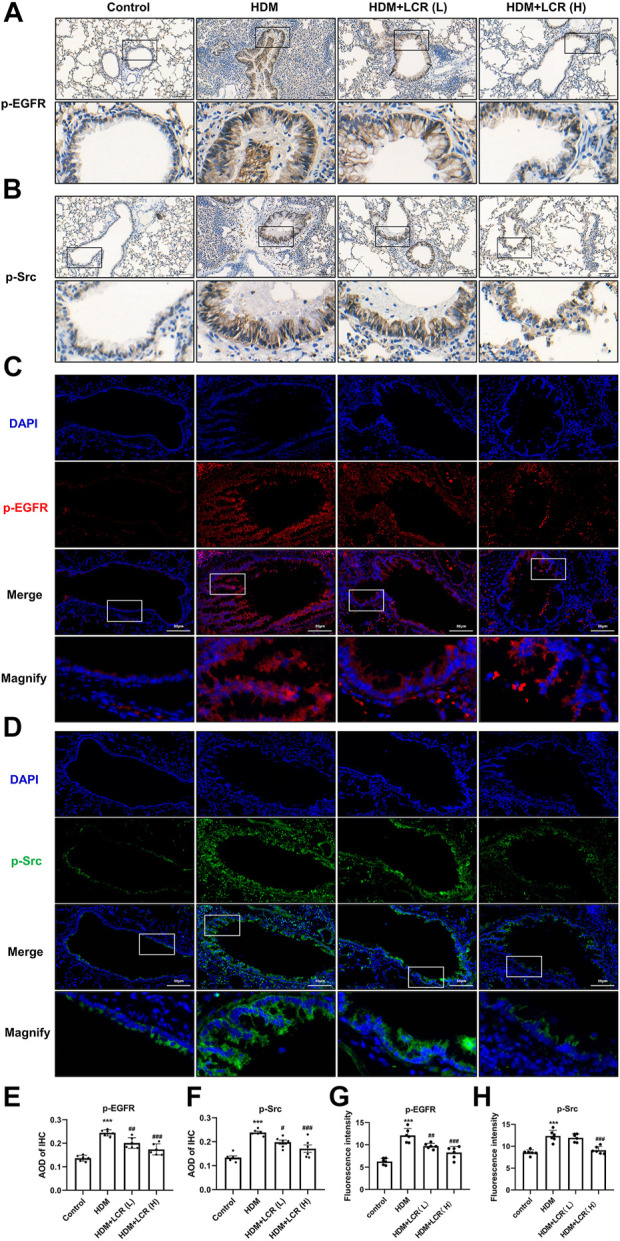
Effects of lonicerin on the immunohistochemistry and immunofluorescence staining of p-EGFR and p-Src. **(A,B)** Immunohistochemical staining of mice lungs for p-Src and p-EGFR. (scale bar, 100 µm). **(C,D)** Immunofluorescent detection of p-Src and p-EGFR in lung sections, p-EGFR was visualized in red, p-Src was visualized in green, and DAPI-stained nuclei was in blue. (scale bar, 50 µm). **(E,F)** Quantitative analysis of immunohistochemical staining for p-Src and p-EGFR by AOD. **(G,H)** Quantitative analysis of immunofluorescence staining for p-Src and p-EGFR by fluorescence intensity. (*n* = 6). Data are expressed as mean ± SEM. Differences between groups were determined with one‐way ANOVA followed by the Tukey’s *post hoc* test. *p < 0.05, **p < 0.01, ***p < 0.001 vs. Control group; #*p* < 0.05, ##*p* < 0.01, ###*p* < 0.001 vs. HDM group.

Further, we detected the localization and expression of p-Src and p-EGFR in lung sections by immunohistochemistry and immunofluorescence. As shown in Figure 8, the results indicated that both p-Src and p-EGFR were predominantly expressed in the airway epithelium. Besides, HDM modeling caused significant upregulation of the AOD values and fluorescence intensity of p-Src and p-EGFR, but these trends were effectively reversed with the use of lonicerin.

Consequently, combined with the enrichment of the EGFR signaling pathway in the KEGG pathway analysis above, we speculate that the possible target suggesting lonicerin for asthma treatment may be Src/EGFR pathway.

## 4 Disscusion

Eosinophilic asthma is the dominant phenotype of asthma, and it was reported that the higher the blood eosinophil count, the worse the prognosis for asthma patients. Patients with eosinophilic asthma are sensitive to glucocorticoid therapy, which is effective in controlling symptoms and suppressing eosinophil levels. However, the side effects of glucocorticoids such as obesity, hypertension, and osteoporosis are of increasing concern (Price et al., 2015; Heaney et al., 2021). Therefore, it is necessary to find safe and effective treatment options for asthma.

Previous studies have found that *Lonicera japonica* extract can treat OVA-induced asthma/allergic rhinitis animal models through the activation of Th1 and Treg cells and inhibition of Th2 and Th17 cells (Hong et al., 2013; Lin et al., 2019; Li et al., 2021). In addition, *Lonicera japonica* extract was found to exert the concentration-dependent bronchial relaxation (Kim et al., 2021). However, the specific components of *Lonicera japonica* that may exert anti-asthmatic effects and the role as well as molecular mechanisms in the HDM-induced asthma model, which could better mimic clinical asthma, have not been investigated. In this study, we found that lonicerin effectively attenuated airway hyperresponsiveness, type 2 inflammatory factor expression, inflammatory cell (especially eosinophil) infiltration, and mucus hypersecretion in HDM-induced eosinophilic asthmatic mice. Airway remodeling has always been a major contradiction in refractory asthma, and it has been reported that biologics and some traditional Chinese medicines could alleviate airway remodeling, but there are still many controversies and lack of long-term clinical observation (Varricchi et al., 2022; Zhou et al., 2022). Our results showed that lonicerin reduced mucus hypersecretion, but cannot reverse collagen fiber deposition, which needs to be further observed with a longer course of medication in future studies.

Network pharmacology analysis was utilized to further explore the potential molecular mechanisms of lonicerin for the treatment of asthma. PPI network analysis identified 10 most strongly interacting hub gene targets: TP53, TNF, SRC, EGFR, KDR, MMP9, BCL2L1, IL2, PTGS2 and MMP2. Interestingly, KEGG pathway enrichment analysis found that the EGFR signaling cascade was one of the most enriched pathways. Therefore, we speculate that lonicerin may exert anti-asthma effects by affecting the EGFR signaling cascade. Further molecular docking analysis revealed that lonicerin had a high binding free energy with EGFR, showing a good binding ability between them. Finally, qPCR and immunohistochemistry verified that EGFR expression and phosphorylation were elevated in the lungs of HDM-induced asthmatic mice, while lonicera significantly reversed these trends.

EGFR is a glycoprotein with a transmembrane tyrosine kinase receptor that is activated by phosphorylation of its ligand binding (Chen et al., 2016). EGFR expression was found to be increased in airway epithelial cells of asthmatic patients, especially those with severe asthma (Amishima et al., 1998). *In vitro* experiments also showed that histamine enhanced EGFR phosphorylation in airway epithelial cells (Hirota et al., 2012). In addition, EGFR expression was upregulated in the lungs of both OVA and HDM-induced asthmatic animals, and treatment with EGFR inhibitors (gefitinib or erlotinib) improved eosinophilic airway inflammation, epithelial barrier function, goblet cell hyperplasia, and AHR (Hur et al., 2007; Jia et al., 2021). These studies implicated that EGFR may be a potential therapeutic target for asthma (Inoue et al., 2020).

Our results also showed that lonicerin not only binded extremely well to Src kinase, but also reversed HDM-induced Src upregulation and phosphorylation. It has been found that Src expression is elevated in asthma animal models and that activation of Src leads to HDM-induced IL-33 secretion as well as activation of type 2 inflammation (Dustin et al., 2021). Knockdown of Src or using the Src inhibitor SU6656 can alleviate airway inflammation in asthmatic animals (El-Hashim et al., 2019; Wu et al., 2022). It is worth mentioning that Src mediates EGFR trans-activation in allergic asthmatic mice, demonstrating that Src is an upstream regulatory molecule of EGFR (Danyal et al., 2016; El-Hashim et al., 2017). Thus, our results suggest that lonicerin may exert anti-asthmatic effects by upregulating the Src/EGFR signaling pathway.

This study has some limitations. Firstly, the databases of network pharmacology are dynamically updated, which may affect the screening of drug targets, and the application of high-throughput omics technology may improve the accuracy. Secondly, it is necessary to explore the downstream regulatory mechanisms of Src/EGFR, the target of lonicerin, in future studies.

In conclusion, this study demonstrated for the first time that lonicerin, a flavonoid natural product derived from *Lonicera japonica*, can reduce airway inflammation, mucus hypersecretion and airway hyperresponsiveness in HDM-induced eosinophilic asthma model. In addition, network pharmacology, molecular docking analysis and experimental validation revealed that the potential target of lonicerin could be the Src/EGFR signaling pathway. Our findings provide a possible avenue for further investigation of the therapeutic role of lonicerin in patients with eosinophilic asthma.

## Data Availability

The datasets presented in this study can be found in online repositories. The names of the repository/repositories and accession number(s) can be found in the article/Supplementary Material.

## Supplementary material

SUPPLEMENTARY FIGURE S1 Effects of lonicerin on HDM-induced deposition of collagen fibers in the lungs. Masson staining was performed to determined collagen deposition. **(B)** The corresponding quantification histograms of **(A)**, respectively. (*n* = 6). Data are expressed as mean ± SEM. Differences between groups were determined with one‐way ANOVA followed by the Tukey’s post-hoc test. ****p* < 0.001 vs. Control group.
